# Improving CT-CBCT deformable image registration for cervical cancer adaptive radiotherapy using a deep learning approach

**DOI:** 10.3389/fonc.2026.1855718

**Published:** 2026-07-06

**Authors:** Chengjian Xiao, Chunlan Huang, Weixiang Lin, Feilong Tian, Youxing Zeng

**Affiliations:** 1 Ganzhou Cancer Hospital, Ganzhou, China; 2 Ganzhou Women and Children’s Health Care Hospital, Ganzhou, China

**Keywords:** adaptive radiotherapy, CBCT, cervical cancer, deep learning, deformable image registration

## Abstract

**Purpose:**

To improve the robustness and anatomical alignment accuracy of CT-CBCT deformable image registration for cervical cancer adaptive radiotherapy.

**Methods:**

A deep learning-based registration framework was developed by enhancing a transformer-based model (UTSRMorph) with a normalized gradient field (NGF) constraint. The network integrates convolutional and transformer modules to capture multi-scale anatomical features. A composite loss function combining mutual information, deformation regularization, and gradient-based similarity was used for training. The method was evaluated on internal and external CBCT-CT datasets using Dice, HD95, and Jacobian determinant.

**Results:**

On the internal dataset, the proposed method achieved Dice scores comparable to the baseline across all structures (bowel: 82.84%), while improving deformation regularity, with low non-positive Jacobian determinant values (%|J|≤0: 0.13), comparable to UTSRMorph (0.12) and lower than VoxelMorph (0.19) and TransMorph (0.21). Slight improvements in Dice and HD95 were observed in bony structures, particularly the hip bones. On the external dataset, the method demonstrated improved generalization, achieving higher Dice for the bowel (83.40% vs. 82.99% and 82.90%) and reduced HD95 (11.70 mm vs. 11.91 mm and 11.85 mm). Improvements were more evident in boundary alignment, especially in high-contrast regions, while maintaining comparable deformation smoothness.

**Conclusion:**

The proposed method (NGF-UTSRMorph) improves the robustness of CT-CBCT registration and enhances boundary alignment without compromising deformation smoothness. Improvements were more evident on the cross-scanner test cohort and in boundary-sensitive metrics, suggesting improved robustness under intensity inconsistencies.

## 1 Introduction

Radiotherapy is one of the primary treatment modalities for locally advanced cervical cancer (1, 2). A typical treatment course spans several weeks, during which substantial and often unpredictable anatomical variations occur due to daily fluctuations in bladder filling, rectal emptying, and tumor regression (3). These variations can significantly alter both the target volume and surrounding organs at risk (OARs), such as the bladder, rectum, and small bowel (4, 5). Dosimetric uncertainties for OARs have been reported to range from 20% to 25% in intra and inter−fraction D2cc values, suggesting that organ deformation contributes significantly to dosimetric errors in OARs (6–8). To ensure accurate dose delivery and optimal sparing of normal tissues, adaptive radiotherapy (ART) has become increasingly important in the management of pelvic malignancies.

Cone-beam computed tomography (CBCT), widely used in image-guided radiotherapy (IGRT), enables daily patient positioning verification and real-time anatomical assessment (9). However, CBCT images typically suffer from severe artifacts, low soft-tissue contrast, and significant intensity inconsistencies compared with planning CT images. These degradations are mainly caused by scatter contamination, beam hardening, and intra-fraction motion (10). Accurately capturing longitudinal anatomical changes using CBCT remains highly challenging. Therefore, developing a robust and efficient deformable image registration (DIR) method for CT-CBCT alignment is essential for dose accumulation, target adaptation, and treatment replanning in ART workflows (11).

Conventional DIR methods, such as optical flow (12), Demons algorithms, and viscous fluid models, rely heavily on intensity-based similarity metrics, including mutual information (MI) and normalized cross-correlation (NCC), to iteratively optimize deformation fields (13, 14). While these methods have been widely applied, they face several limitations in CT-CBCT registration. First, substantial intensity discrepancies and imaging artifacts in CBCT often lead to unreliable voxel-wise correspondence and suboptimal convergence. Second, repeated spatial regularization tends to produce overly smooth deformation fields, limiting the ability to capture complex local deformations in pelvic organs (15). Finally, their high computational cost makes them unsuitable for time-sensitive clinical scenarios such as online ART. Recently, deep learning-based registration methods have demonstrated great potential in overcoming these limitations by learning direct mappings between image pairs, enabling fast and end-to-end inference (16–18). In particular, unsupervised frameworks based on spatial transformer networks (STNs) have become the dominant paradigm due to the lack of ground-truth deformation fields (19). Among these approaches, convolutional neural network (CNN)-based models, such as VoxelMorph (20), have shown promising performance in medical image registration tasks. However, CNNs are inherently limited by their local receptive fields, making it difficult to model long-range spatial dependencies required for capturing large and complex deformations (21). In pelvic radiotherapy, organ motion caused by variations in bladder and bowel filling can induce substantial displacement of the uterus and cervix, posing significant challenges for CNN-based registration models. To address this issue, Vision Transformers (ViTs), particularly hierarchical architectures such as Swin Transformer, have been introduced into medical image registration to capture global contextual information through self-attention mechanisms (22, 23). By modeling long-range dependencies and multi-scale features, transformer-based approaches provide a promising solution for handling large anatomical variations in CT-CBCT registration. Zhang et al. (24) proposed UTSRMorph, a lightweight hybrid framework that employs a super-resolution (SR) decoder to generate high-resolution deformation fields from multi-scale features, achieving strong performance in deformable registration. Despite these advances, a critical challenge remains in CT-CBCT deformable registration: the reliability of similarity measurements under severe intensity inconsistencies. Most existing methods rely on intensity-based similarity metrics, such as NCC or mean squared error (MSE), which assume consistent intensity distributions between images. However, this assumption is often violated in CBCT due to noise, artifacts, and reconstruction differences. Consequently, these methods may produce inaccurate alignment, particularly in low-contrast soft-tissue regions where intensity cues are weak and ambiguous. This limitation is especially problematic in pelvic imaging, where accurate alignment of deformable structures, such as the small bowel, is crucial for reliable dose estimation.

To overcome the limitations of intensity-based similarity measures, gradient-based approaches have been proposed as a more robust alternative. The normalized gradient field (NGF) (25) focuses on aligning gradient orientations rather than raw intensity values, making it less sensitive to modality-specific intensity variations and more effective in capturing structural information. By emphasizing edge and boundary consistency, NGF is suitable for multimodal or low-contrast image registration tasks, such as CT-CBCT alignment. However, its integration into modern transformer-based registration frameworks remains underexplored. Motivated by the above challenges, we aim to improve the robustness of CT-CBCT deformable registration under severe intensity inconsistencies. We build upon a strong transformer-based registration framework, UTSRMorph, which has demonstrated effectiveness in modeling large and complex deformations. However, despite its powerful representation capability, UTSRMorph still relies on conventional intensity-based similarity metrics during training, which limits its performance in CBCT scenarios where intensity distributions are highly inconsistent and unreliable. To address this limitation, we introduce a gradient-based similarity constraint based on normalized gradient fields (NGF) into the training process. By emphasizing structural consistency rather than raw intensity matching, the proposed approach enhances boundary alignment and improves robustness in low-contrast regions.

## 2 Materials and methods

**Figure 1:**
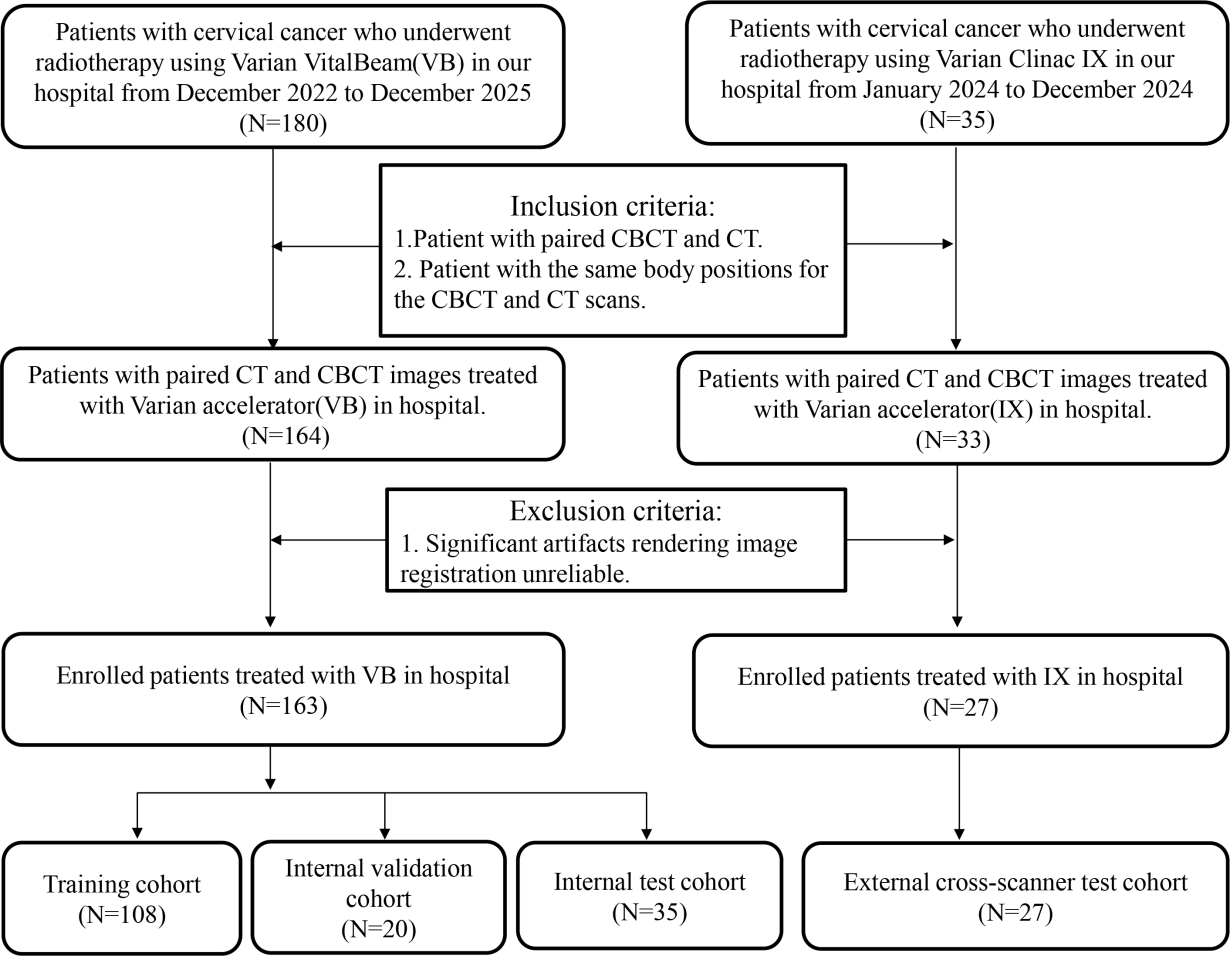
The overview of data acquisition and processing.

**Figure 2:**
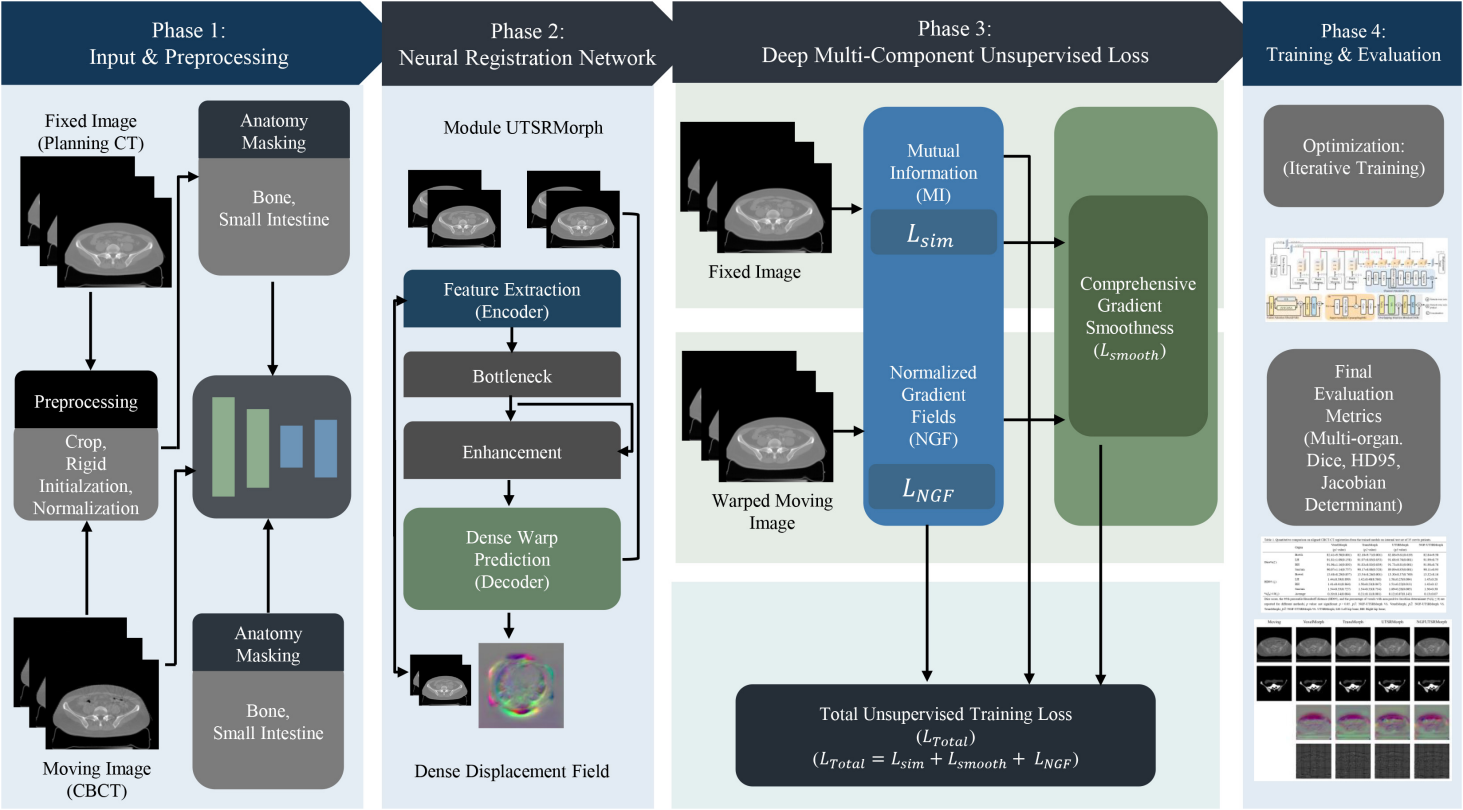
Schematic workflow of the proposed unsupervised deep learning registration framework. The pipeline comprises: image preprocessing, and an unsupervised loss formulation integrating Mutual Information (MI), Normalized Gradient Fields (NGF), and smoothness constraints. Final performance is validated using multi-organ anatomical metrics.

The overall workflow of the proposed framework, including data acquisition and processing, is illustrated in **Figure 1** and **Figure 2**. This study was conducted on a cervical cancer CT-CBCT dataset collected from different scanners within the same institution. The internal cohort consisted of 163 patients acquired from the author’s hospital using a Varian linear accelerator-VitalBeam(Varian Medical Systems, Palo Alto, CA, USA). The dataset was split into 108 patients for training, 20 for validation, and 35 for internal testing. To evaluate model robustness under scanner-related domain shift, an independent cross-scanner test cohort comprising 27 patients was collected from another Varian accelerator (Clinac IX) within the same institution.For all patients, planning CT (pCT) and corresponding fraction CBCT images were acquired. The pCT images were used as fixed volumes, while CBCT images were treated as moving volumes. All pCT images were obtained using a Philips Brilliance Big Bore CT scanner (Philips Medical Systems, Netherlands) with a tube voltage of 120 kVp, tube current of 320 mA, and slice thickness of 5 mm. The reconstructed pCT image size ranged from 512 × 512 × 80 to 150. The CBCT images were acquired using onboard cone-beam CT systems integrated with Varian linear accelerators. For the internal cohort (VitalBeam), CBCT acquisition was performed using 125 kVp and 60 mA, whereas the cross-scanner test cohort acquired on Clinac IX used 125 kVp and 80 mA. All CBCT images had a slice thickness of 2 mm and a reconstructed image size of 512 × 512 × 88. Before deformable registration, all CT-CBCT image pairs were first rigidly aligned to reduce global positional differences between the planning CT and CBCT images. The rigidly aligned image pairs were subsequently used as inputs for all deformable registration methods. To ensure spatial consistency, all CT and CBCT volumes were resampled to an isotropic voxel spacing of 0.908 × 0.908 × 1.98 mm³. Due to memory constraints and to focus on the pelvic region of interest, all volumes were cropped or padded to a fixed size of 384 × 384 × 96. Intensity values were normalized to [0, 1]. Manual segmentations were performed by experienced radiation oncologists and used as ground truth for evaluation. Four key anatomical structures relevant to cervical cancer radiotherapy were included: the small bowel, left hip bone, right hip bone, and sacrum. For the internal and external test set, all four structures were used for quantitative evaluation, evaluation was primarily focused on the bowel and bony structures (hip bones and sacrum), depending on image quality and visibility.

The proposed NGF-UTSRMorph and all comparison methods were implemented in PyTorch 1.12. Training was performed on an NVIDIA L20 GPU (48 GB). The Adam optimizer was used with an initial learning rate of 2 × 10^−4^. The batch size was set to 1 due to memory constraints. All models were trained for 200 epochs, and the optimal model was selected based on the highest average Dice score on the validation set.

## 3 Network architecture

**Figure 3:**
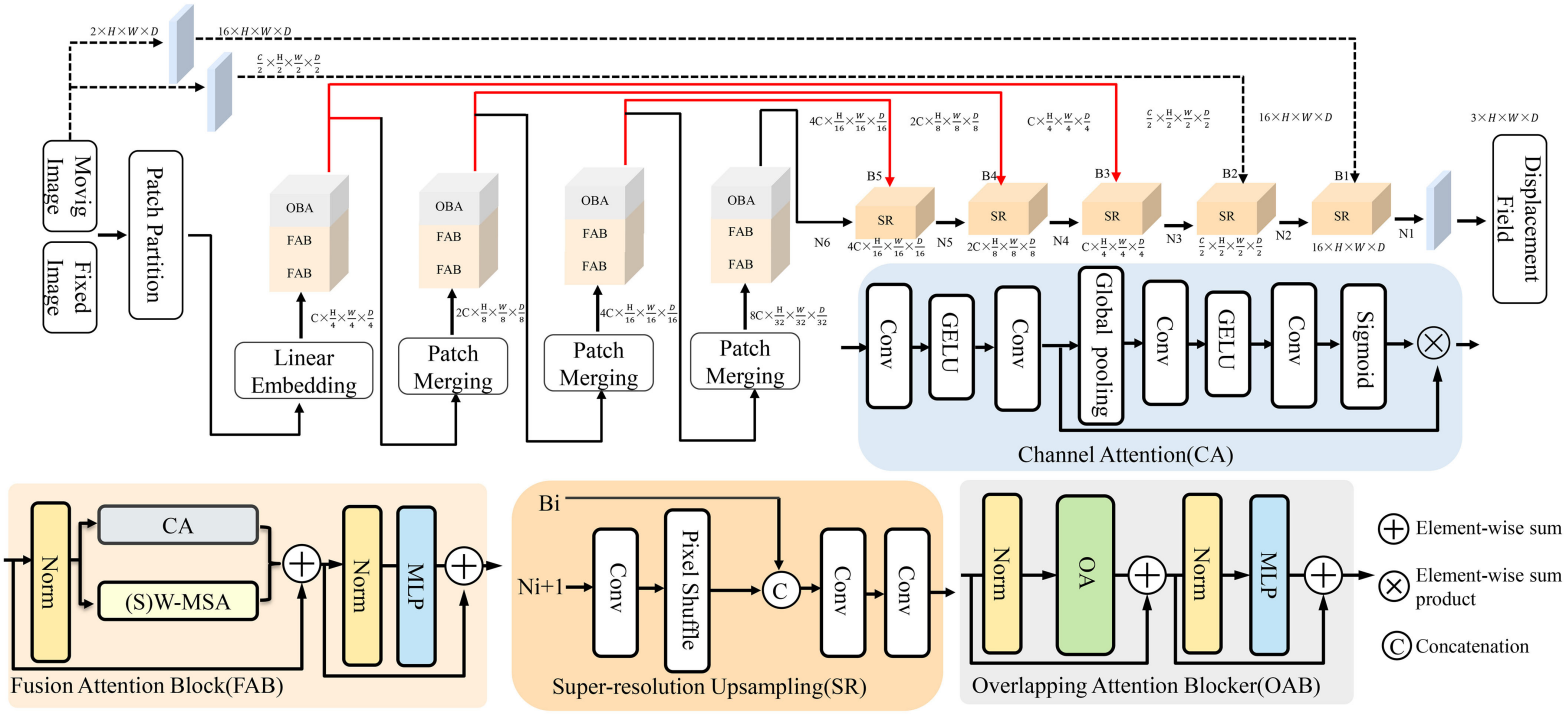
The UTSRMorph architecture extends the unified transformer and super-resolution network. The encoder comprises four hierarchical stages, each consisting of Fusion Attention Blocks (FABs) and an Overlapping Attention Block (OAB) to capture multi-scale spatial dependencies.

UTSRMorph is a unified Transformer-based and super-resolution-driven framework for unsupervised deformable medical image registration. The overall architecture of the proposed method is illustrated in **Figure 3**. It adopts a U-Net-like encoder-decoder structure, in which the encoder jointly leverages the complementary strengths of convolutional neural networks (CNNs) and Vision Transformers (ViTs), while the decoder formulates displacement field generation as a super-resolution reconstruction task. Given a fixed image *f* and a moving image *m*, the two volumes are concatenated along the channel dimension and fed into the network for feature extraction. The encoder learns hierarchical multi-scale feature representations that capture both local structural details and long-range spatial dependencies, while the decoder progressively reconstructs a dense, high-resolution displacement field.

The encoder consists of four hierarchical stages. Inspired by the Swin Transformer architecture, the input 3D volume is first partitioned into non-overlapping patches, which are then linearly projected into token embeddings. As the network deepens, spatial resolution is progressively reduced via patch merging, while the feature dimensionality is increased to enhance representation capacity. Each stage comprises multiple Fusion Attention Blocks (FABs) followed by an Overlapping Attention Block (OAB), enabling effective integration of local and global contextual information. Specifically, the FAB combines window-based multi-head self-attention with a convolutional channel attention (CA) mechanism. The self-attention module captures long-range contextual dependencies within local windows, while the channel attention module aggregates global spatial information through convolutional operations and adaptively recalibrates channel-wise feature responses. This hybrid design enhances feature discrimination and suppresses redundant information, leading to more robust feature representations.

To further strengthen spatial correspondence modeling, the OAB introduces a cross-attention mechanism with overlapping windows. Unlike standard Swin Transformer blocks, where attention is computed within non-overlapping windows, OAB generates query features from non-overlapping windows while deriving key and value features from larger overlapping regions. This design effectively enlarges the receptive field and allows each token to access richer contextual information beyond strict window boundaries, thereby improving feature continuity and correspondence learning across regions. In the decoder, displacement field estimation is formulated as a super-resolution problem. Instead of relying on conventional interpolation-based up-sampling, a learnable super-resolution module based on sub-pixel convolution is employed. At each decoding stage, low-resolution feature maps are first expanded along the channel dimension and then rearranged spatially to reconstruct high-resolution feature representations. Skip connections are introduced to fuse encoder and decoder features at corresponding scales, facilitating effective multi-scale information integration and mitigating information loss during decoding. This design enables the network to generate smooth and spatially detailed deformation fields. The final output of the network is a dense displacement field *ϕ*, which is applied to the moving image through a spatial transformer layer to produce the warped image *m* ○ *ϕ*.

To further improve structural alignment, particularly at anatomical boundaries, a gradient-based similarity constraint is incorporated during training. A NGF loss is introduced to encourage the alignment of local image gradient orientations between the fixed and warped moving images. This constraint complements conventional intensity-based similarity measures by emphasizing structural consistency rather than absolute intensity matching. Such a design is particularly advantageous for CT-CBCT registration, where intensity discrepancies and low soft-tissue contrast are common, allowing the model to better capture boundary information and improve alignment robustness.

## 4 Loss functions

The training process is guided by a composite loss function that ensures both accurate image alignment and physically plausible deformation fields. A MI loss with a weight of 1 is adopted for the cervical cancer CBCT-CT dataset. Additionally, a loss term is incorporated with a weighting factor λ = α = 1; The objective function is defined as in Equation 1:

(1) $$L\left(f,m,\emptyset \right)=L_{sim}\left(f,m\;○\;\emptyset \right)+\lambda L_{Smooth}\left(\emptyset \right)\;+\alpha L_{NGF}$$

λ and *α* denotes the regularization weight.

1) Image Similarity Loss: The similarity loss L_sim_ is defined as the voxel-wise MI between the fixed image *f* and the warped moving image *m* ○ Ø. The MI between two random variables *X, Y* can be defined in Equation 2:

(2) $$MI\left(X,Y\right)=\iint p\left(x,y\right)\log\frac{p\left(x,y\right)}{p\left(x\right)p\left(y\right)}dxdy$$

where *p*(*x*, *y*), *p*(*x*), *p*(*y*) are probability density functions. MI is integrated to capture statistical dependencies between heterogeneous intensity distributions. To address this, researchers have proposed various approaches to estimate and optimize its calculation. For instance, the Parzen window density estimation method (26) is utilized to derive the probability density and subsequently calculate MI.

2) Deformation Field Regularization Loss: Optimizing *L_sim_* may cause Ø acute deformation with unrealistic physical motion. Therefore, a diffusion-based regularization term is introduced as shown in Equation 3:

(3) $$L_{Smooth}\left(\phi \right)=\sum_{P\epsilon \text{Ω}}\nabla u{\left(P\right)}^{2}$$

where *u* is the spatial gradient of the displacement field.

3) The NGF loss is defined in Equation 4:

(4) $$L_{NGF\left(x\right)}=\frac{1}{|\text{Ω}|}\sum_{x\in \text{Ω}}\left(1-\frac{{\left(\nabla I\left(x\right)\cdot \nabla J\left(x\right)\right)}^{2}}{∥\nabla I\left(x\right){∥}^{2}\cdot ∥\nabla J\left(x\right){∥}^{2}+\epsilon }\right)$$

where ∇ denotes the spatial gradient operator, ⟨·⟩ represents the dot product, and *ϵ* is a small constant for numerical stability. Unlike intensity-based similarity measures, NGF focuses on aligning gradient orientations rather than raw intensities, making it less sensitive to modality-specific intensity differences. This property is particularly advantageous for CT-CBCT registration, where soft-tissue contrast is limited and structural boundaries provide more reliable alignment cues.

## 5 Evaluation metrics

To quantitatively evaluate the performance of the proposed registration framework, we adopted widely used metrics in medical image registration, including the Dice Similarity Coefficient (DSC), the 95th percentile Hausdorff Distance (HD95), and Jacobian determinant–based measurements.

The DSC measures the spatial overlap between the warped segmentation and the ground-truth segmentation in Equation 5:

(5) $$Dice\left(X,Y\right)=\frac{2\left|X\cap Y\right|}{\left|X\right|+\left|Y\right|}$$

where *X* denotes the warped binary segmentation mask (prediction), and *Y* denotes the ground-truth segmentation mask of the fixed image.

To quantify the boundary alignment between the fixed and warped segmentations, we employ the 95th percentile HD95. Unlike the standard HD, which is highly sensitive to isolated surface points, HD95 offers a more robust characterization of structural correspondence by mitigating the influence of extreme outliers. The definition of HD is written as Equation 6:

(6) $$HD\left(S_{F},S_{W}\right)=max\left(\underset{P_{W}\in \Omega_{W}}{\max}d\left(P_{W},\Omega_{F}\right),\underset{P_{F}\in \Omega_{F}}{\max}d\left(P_{F},\Omega_{W}\right)\right)$$

where Ω*_F_*, Ω*_W_* refers to all surface voxels of the fixed label image and the segmentation of the warped image. *P_F_*, *P_W_* refers to an arbitrary voxel in Ω*_F_*, Ω*_W_*. *d*(*P_F_*, Ω*_Q_*) represents the shortest path from point *P_F_* on the surface of the fixed label image to the surface of the warped segmentation image. The same definition applied for *d*(*P_W_*, Ω*_F_*).

To assess deformation regularity, we compute the percentage of voxels with non-positive Jacobian determinants ( $\text{det}\left(J_{\phi }\right)\leq 0$), which indicate non-diffeomorphic transformations (folding). Lower values correspond to better topology preservation. We computed the percentage of voxels with non-positive Jacobian determinants: %|Jϕ ≤ 0|. A lower percentage indicates better topological preservation and smoother deformation fields. All metrics were computed separately for the bowel, left hip bone, right hip bone, and sacrum. Results are reported as mean ± standard deviation.

## 6 Statistical analysis

All statistical analyses were performed using Python (v3.10.13) and SPSS Statistics 21. Paired comparisons were conducted using the Wilcoxon signed-rank test. A *p*-value< 0.05 was considered statistically significant. Given the exploratory nature of this study involving multiple pairwise comparisons across organs, metrics, and datasets, no formal correction for multiple comparisons was applied. Therefore, the reported *p*-values should be interpreted with appropriate caution.

## 7 Results

### 7.1 Evaluation on the internal test set

**Figure 4:**
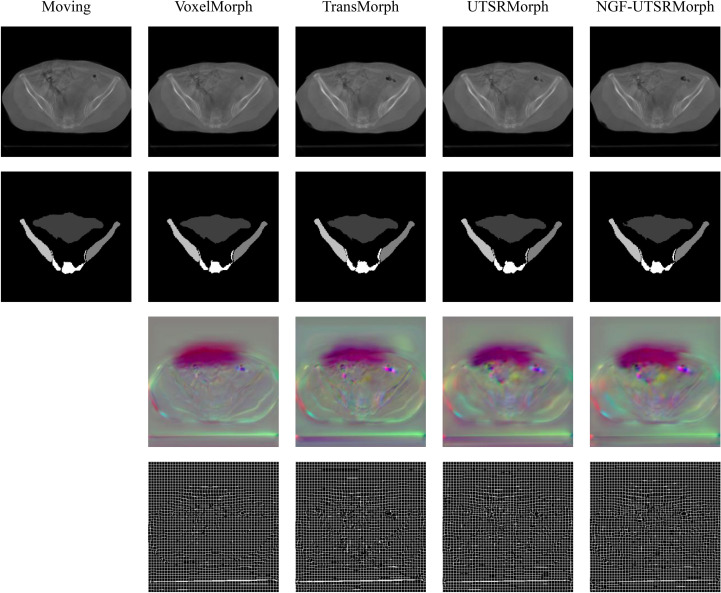
Qualitative results of different registration methods of CT-CBCT registration on the internal cervical cancer test set. The first row displays the deformed moving images, the second row displays deformed OAR masks including the bowel, left hip bone, right hip bone, and Sacrum, the third row visualizes the deformation fields, and the last row illustrates the deformed grids.

The quantitative results on the internal test cohort of 35 cervix patients are summarized in **Table 1**, while qualitative comparisons are presented in **Figure 4**. Overall, the proposed NGF-UTSRMorph achieved competitive performance across all evaluated organs while maintaining improved deformation regularity. In terms of Dice similarity coefficient, NGF-UTSRMorph yielded 82.84 ± 9.50% for the bowel, which is slightly higher than VoxelMorph (82.41 ± 9.50%, *p* < 0.001) and TransMorph (82.10 ± 9.71%, *p* < 0.001), and comparable to UTSRMorph (82.88 ± 9.61%, *p* = 0.619). For bony structures, NGF-UTSRMorph achieved the highest Dice score for the left hip (91.99 ± 0.75%), showing no significant difference compared with VoxelMorph (*p* = 0.158) and TransMorph (*p* = 0.853), but a significant improvement over UTSRMorph (*p* < 0.001). A similar trend was observed for the right hip, where NGF-UTSRMorph reached 91.98 ± 0.76%, significantly outperforming TransMorph (*p* = 0.039) and UTSRMorph (*p* < 0.001). For the sacrum, all methods achieved comparable performance, with NGF-UTSRMorph obtaining 90.11 ± 0.93%.

**Table 1:** Quantitative comparison on aligned CBCT-CT registration from the trained models on internal test set of 35 cervix patients.

	Organ	VoxelMorph (*p1* value)	TransMorph (*p2* value)	UTSRMorph (*p3* value)	NGF-UTSRMorph
Dice% (↑)	Bowel	82.41 ± 9.50 (0.001)	82.10 ± 9.71 (0.001)	82.88 ± 9.61 (0.619)	82.84 ± 9.50
	LH	91.81 ± 1.09 (0.158)	91.97 ± 0.85 (0.853)	91.68 ± 0.76 (0.001)	91.99 ± 0.75
	RH	91.96 ± 1.16 (0.893)	91.83 ± 0.83 (0.039)	91.73 ± 0.81 (0.001)	91.98 ± 0.76
	Sacrum	90.07 ± 1.14 (0.757)	90.17 ± 0.88 (0.528)	89.89 ± 0.85 (0.001)	90.11 ± 0.93
HD95 (↓)	Bowel	13.48 ± 8.29 (0.057)	13.54 ± 8.26 (0.001)	13.30 ± 8.37 (0.769)	13.32 ± 8.16
	LH	1.44 ± 0.39 (0.899)	1.42 ± 0.40 (0.566)	1.56 ± 0.25 (0.004)	1.45 ± 0.26
	RH	1.41 ± 0.41 (0.864)	1.50 ± 0.23 (0.047)	1.51 ± 0.22 (0.013)	1.42 ± 0.12
	Sacrum	1.54 ± 0.35 (0.727)	1.54 ± 0.33 (0.734)	1.69 ± 0.28 (0.005)	1.56 ± 0.30
%| *J_θ_* |≤0 (↓)	Average	0.19 ± 0.14 (0.004)	0.21 ± 0.11 (0.001)	0.12 ± 0.07 (0.143)	0.13 ± 0.07

Dice score, the 95th percentile Hausdorff distance (HD95), and the percentage of voxels with non-positive Jacobian determinant (%|J_θ_| ≤ 0) are reported for different methods; *p* value: not significant: *p*>0.05. *p1*, NGF-UTSRMorph vs. VoxelMorph; *p2*, NGF-UTSRMorph vs. TransMorph; *p3*, NGF-UTSRMorph vs. UTSRMorph; LH, Left hip bone; RH, Right hip bone.

↑ means higher is better and ↓ means lower is better.

NGF-UTSRMorph demonstrated consistently improved or comparable HD95 values. Specifically, the HD95 for the bowel was reduced to 13.32 ± 8.16 mm, lower than VoxelMorph (13.48 ± 8.29 mm) and TransMorph (13.54 ± 8.26 mm), and comparable to UTSRMorph (13.30 ± 8.37 mm, *p* = 0.769). For the hip bones, NGF-UTSRMorph achieved 1.45 ± 0.26 mm for the left hip and 1.42 ± 0.12 mm for the right hip, with the latter showing significant improvements over TransMorph (*p* = 0.047) and UTSRMorph (*p* = 0.013). For the sacrum, NGF-UTSRMorph obtained 1.56 ± 0.30 mm, which is slightly higher than VoxelMorph but significantly lower than UTSRMorph (*p* = 0.005). In addition, NGF-UTSRMorph substantially improved the topology of the deformation field. The percentage of voxels with non-positive Jacobian determinant was reduced to 0.13 ± 0.07, compared to 0.19 ± 0.14 for VoxelMorph (*p* = 0.004) and 0.21 ± 0.11 for TransMorph (*p* < 0.001), and remained comparable to UTSRMorph (0.12 ± 0.07, *p* = 0.143), indicating more physically plausible deformations.

### 7.2 Evaluation on the cross-scanner test cohort

**Figure 5:**
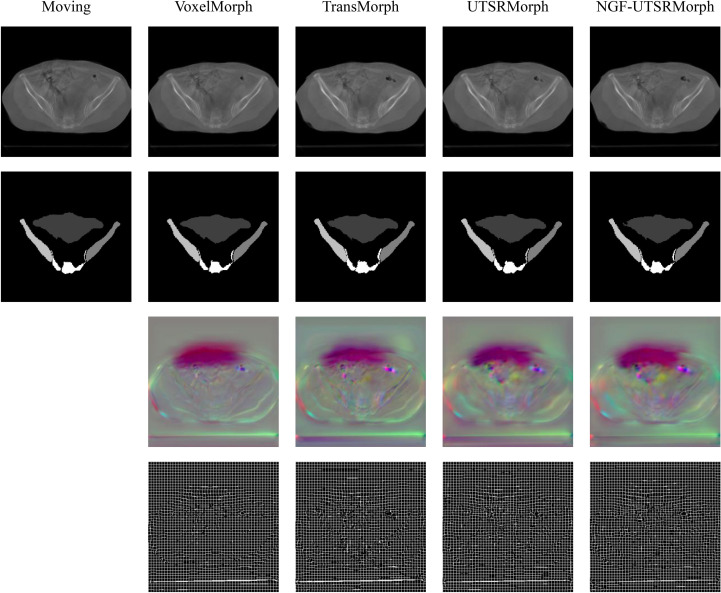
Qualitative results of different registration methods of CT-CBCT registration on evaluation on the cross-scanner test cohort. The first row displays the deformed moving images, the second row displays deformed OAR masks including the bowel, left hip bone, right hip bone, and Sacrum, the third row visualizes the deformation fields, and the last row illustrates the deformed grids.

The results on the independent cross-scanner test cohort of 27 patients are presented in **Table 2** and **Figure 5**. NGF-UTSRMorph demonstrated improved generalization capability, particularly for soft tissue structures. For the bowel, NGF-UTSRMorph achieved the highest Dice score of 83.40 ± 6.79%, significantly outperforming VoxelMorph (82.99 ± 7.00%, *p* = 0.018), TransMorph (82.90 ± 6.72%, *p* = 0.005), and UTSRMorph (83.17 ± 6.83%, *p* = 0.020). For the left hip, NGF-UTSRMorph achieved 92.42 ± 1.00%, which is comparable to VoxelMorph (92.55 ± 1.02%, *p* = 0.129) and TransMorph (92.47 ± 1.00%, *p* = 0.449), while significantly outperforming UTSRMorph (92.00 ± 1.02%, *p* < 0.001). For the right hip, NGF-UTSRMorph obtained 92.18 ± 0.94%, showing significant improvements over TransMorph (p< 0.001) and UTSRMorph (*p* < 0.001). In contrast, for the sacrum, NGF-UTSRMorph achieved 76.18 ± 9.19%, which is comparable to VoxelMorph (*p* = 0.992) and UTSRMorph (*p* = 0.119), but slightly lower than TransMorph (77.98 ± 7.88%, *p* = 0.003).

**Table 2:** Quantitative comparison on aligned CBCT-CT registration from the trained models on external test set of 27 cervix patients.

	Organ	VoxelMorph (*p1* value)	TransMorph (*p2* value)	UTSRMorph (*p3* value)	NGF-UTSRMorph
Dice% (↑)	Bowel	82.99 ± 7.00 (0.018)	82.90 ± 6.72 (0.005)	83.17 ± 6.83 (0.020)	83.40 ± 6.79
	LH	92.55 ± 1.02 (0.129)	92.47 ± 1.00 (0.449)	92.00 ± 1.02 (0.001)	92.42 ± 1.00
	RH	92.49 ± 1.08 (0.007)	92.46 ± 0.96 (0.001)	91.92 ± 1.14 (0.001)	92.18 ± 0.94
	Sacrum	76.18 ± 8.90 (0.992)	77.98 ± 7.88 (0.003)	76.94 ± 8.37 (0.119)	76.18 ± 9.19
HD95 (↓)	Bowel	11.91 ± 6.32 (0.004)	11.85 ± 6.22 (0.084)	11.79 ± 6.69 (0.132)	11.70 ± 6.31
	LH	1.33 ± 0.29 (0.183)	1.37 ± 0.24 (0.467)	1.55 ± 0.29 (0.002)	1.40 ± 0.28
	RH	1.40 ± 0.29 (0.057)	1.42 ± 0.24 (0.028)	1.53 ± 0.24 (0.675)	1.52 ± 0.34
	Sacrum	5.12 ± 2.99 (0.467)	4.79 ± 2.92 (0.003)	4.98 ± 2.97 (0.146)	5.08 ± 2.97
%| *J_θ_* |≤0 (↓)	Average	0.08 ± 0.05 (0.361)	0.10 ± 0.04 (0.002)	0.06 ± 0.04 (0.001)	0.08 ± 0.05

Dice score, the 95th percentile Hausdorff distance (HD95), and the percentage of voxels with non-positive Jacobian determinant (%|J_θ_| ≤ 0) are reported for different methods. *p* value: not significant: *p*>0.05. *p1*, NGF-UTSRMorph vs. VoxelMorph; *p2*, NGF-UTSRMorph vs. TransMorph; *p3*, NGF-UTSRMorph vs. UTSRMorph; LH, Left hip bone; RH, Right hip bone.

↑ means higher is better and ↓ means lower is better.

In terms of HD95, NGF-UTSRMorph achieved the best performance for the bowel with 11.70 ± 6.31 mm, which is significantly lower than VoxelMorph (11.91 ± 6.32 mm, *p* = 0.004) and slightly lower than TransMorph and UTSRMorph without statistical significance. For the left hip, NGF-UTSRMorph (1.40 ± 0.28 mm) showed comparable performance to other methods, while for the right hip and sacrum, the differences across methods were relatively small and did not show consistent trends. Regarding deformation regularity, NGF-UTSRMorph achieved 0.08 ± 0.05, which is comparable to VoxelMorph (*p* = 0.361) and significantly better than TransMorph (*p* = 0.002), although slightly higher than UTSRMorph (0.06 ± 0.04, *p* < 0.001).

## 8 Discussion

In this study, we enhanced a transformer-based deformable registration framework for CT-CBCT alignment in cervical cancer radiotherapy by incorporating a gradient-based similarity constraint. Quantitative and qualitative results showed that the proposed NGF-UTSRMorph achieved stable but relatively modest improvements across different anatomical structures and evaluation metrics. Compared with the baseline UTSRMorph, the proposed method mainly improved boundary-sensitive metrics and demonstrated better robustness on the cross-scanner test cohort, while global overlap metrics such as Dice remained largely comparable.

The comparison between UTSRMorph and NGF-UTSRMorph constitutes a controlled evaluation, as both models share the same architecture and training configuration, differing only in the inclusion of the NGF loss term. On the internal test set, NGF-UTSRMorph achieved Dice scores comparable to UTSRMorph, whereas improvements were more evident in boundary-based metrics such as HD95, particularly on the cross-scanner test cohort. These findings suggest that the NGF constraint mainly contributes to boundary refinement and robustness under intensity inconsistencies rather than substantially altering global volumetric overlap. This behavior may be attributed to the strong baseline performance of the transformer-based architecture, where further improvements in Dice become less pronounced. In addition, Dice is relatively insensitive to local boundary refinement and may not fully capture subtle structural improvements.

From a methodological perspective, the observed improvements can be explained by the complementary characteristics of MI and NGF. MI captures global statistical dependencies between multimodal images, whereas NGF emphasizes local structural consistency through gradient orientation alignment. Since NGF is less sensitive to intensity inconsistencies, it is suitable for CT-CBCT registration. Their combination therefore enables improved boundary delineation while preserving global alignment. In this study, the weighting factor α was empirically set to 1 to balance the contributions of the MI, smoothness regularization, and NGF terms. This setting demonstrated stable performance across both the internal and cross-scanner cohorts. Nevertheless, a dedicated sensitivity analysis may provide further insight into the optimal weighting strategy and will be explored in future work.

Structure-specific analysis further revealed that the effectiveness of NGF depends on image characteristics. In high-contrast regions such as pelvic bones, reliable gradient information enables more effective boundary refinement, leading to consistent improvements in HD95 and, in some cases, Dice scores. In contrast, deformable soft tissues such as the bowel often exhibit low contrast and ambiguous boundaries on CBCT, resulting in less reliable gradient information and therefore more limited improvements on the internal dataset. However, on the cross-scanner test cohort, where intensity inconsistencies were more pronounced, NGF contributed to improved robustness, particularly for bowel registration. Another important finding is that the proposed method maintained deformation regularity while improving alignment accuracy. The percentage of voxels with non-positive Jacobian determinants remained low across both datasets, indicating that incorporation of NGF did not introduce additional folding or instability. This suggests that the proposed framework achieves a favorable balance between registration accuracy and deformation smoothness.

From a clinical perspective, improved boundary alignment may still be relevant for adaptive radiotherapy workflows, particularly for contour propagation and dose accumulation (27). Nevertheless, several limitations should be acknowledged. First, evaluation mainly focused on bowel and bony structures because of the availability and consistency of retrospective annotations. Clinically important pelvic soft-tissue structures, including the bladder, rectum, and cervix, were not comprehensively evaluated. Future work will incorporate additional pelvic organs, target volumes, and dosimetric evaluation to further assess clinical utility. Second, the effectiveness of NGF remained limited in low-contrast soft-tissue regions, suggesting that additional strategies such as multi-scale gradient modeling may further improve performance. Third, this study focused primarily on geometric evaluation metrics and did not include direct dosimetric analysis such as DVH or dose accumulation assessment. In addition, landmark-based evaluation metrics such as target registration error (TRE) were not included because reliable anatomical landmark annotations were unavailable in the retrospective CT-CBCT dataset. Clinician-based qualitative assessment was also not performed and will be investigated in future work. Finally, inference efficiency was not comprehensively evaluated. Since the proposed NGF constraint is incorporated only during training and does not alter the inference architecture, the computational complexity is expected to remain comparable to the baseline UTSRMorph framework.

## 9 Conclusion

The proposed NGF-UTSRMorph framework improves boundary alignment robustness and maintains stable deformation regularity in CT-CBCT deformable registration, particularly on the cross-scanner test cohort. These findings suggest that gradient-based structural constraints can effectively complement transformer-based registration frameworks for cervical cancer adaptive radiotherapy applications.

## Data Availability

The imaging datasets analyzed in this study are not publicly available to protect patient privacy. De-identified data are available from the corresponding author upon reasonable request and with approval from the institutional ethics committee.

## References

[1] Fahmi MN Kusuma F Hellyanti T . High ALDH-1 expression predicts non-complete response of radiotherapy in stage III squamous cell cervical carcinoma patients. Asian Pacific J Cancer Prev. (2023) 24:1863–8. doi: 10.31557/apjcp.2023.24.6.1863 37378913 PMC10505876

[2] Palani D Govindaraj K Sampathrajan S Karunagaran L Ganesh KM . A dosimetric analysis of modified volumetric modulated arc therapy for bone marrow sparing radiotherapy in cervical cancer-an alternative approach to conventional VMAT. Asian Pac J Cancer Prev. (2022) 23:4323–32. doi: 10.31557/APJCP.2022.23.12.4323 36580016 PMC9971454

[3] Wang E Yen A Hrycushko B Wang S Lin J Zhong X . The accuracy of artificial intelligence deformed nodal structures in cervical online cone-beam-based adaptive radiotherapy. Phys Imaging Radiat Oncol. (2024) 29:100546. doi: 10.1016/j.phro.2024.100546 38369990 PMC10869256

[4] Yan D . Adaptive radiotherapy: merging principle into clinical practice. Semin Radiat Oncol. (2010) 20:79–83. doi: 10.1016/j.semradonc.2009.11.001 20219545

[5] Thörnqvist S Hysing LB Tuomikoski L Vestergaard A Tanderup K Muren LP . Adaptive radiotherapy strategies for pelvic tumors – a systematic review of clinical implementations. Acta Oncol. (2016) 55(8):943–58. doi: 10.3109/0284186X.2016.1156738 27055486

[6] Dinkla AM Pieters BR Koedooder K Meijnen P Van Wieringen N Rob VDL . Deviations from the planned dose during 48 hours of stepping source prostate brachytherapy caused by anatomical variations. Radiother Oncol. (2013) 107:106–11. doi: 10.1016/j.radonc.2012.12.011 23333013

[7] Lobefalo F Bignardi M Reggiori G Tozzi A Tomatis S Alongi F . Dosimetric impact of inter-observer variability for 3D conformal radiotherapy and volumetric modulated arc therapy: the rectal tumor target definition case. Radiat Oncol. (2013) 8:176. doi: 10.1186/1748-717x-8-176 23837942 PMC3720254

[8] Kirisits C Tanderup K Nesvacil N Pötter R . Uncertainties in image guided adaptive cervix cancer brachytherapy: impact on planning and prescription. Radiother Oncol. (2013) 107:1–5. doi: 10.1016/j.radonc.2013.02.014 23541642

[9] Terhaard CHJ . Image-guided radiotherapy. Dtsch Aerzteblatt Int. (2004) 32:33–7.

[10] Zachiu C De Senneville BD Tijssen RHN Kotte ANTJ Houweling AC Kerkmeijer LGW . Non-rigid CT/CBCT to CBCT registration for online external beam radiotherapy guidance. Phys Med Biol. (2017) 62(17):6877–96. doi: 10.1088/1361-6560/aa7d19 29116054

[11] Kim H Park SB Monroe JI Traughber BJ Zheng Y Lo SS . Quantitative analysis tools and digital phantoms for deformable image registration quality assurance. Technol Cancer Res Treat. (2015) 14:428–39. doi: 10.1177/1533034614553891 25336380

[12] Østergaard Noe K De Senneville BD ElstrøM UV Tanderup K S?Rensen TS . Acceleration and validation of optical flow based deformable registration for image-guided radiotherapy. Acta Oncol (Madr). (2017) 47:1286–93. doi: 10.1080/02841860802258760 18661435

[13] Andronache A Siebenthal MV Székely G Cattin P . Non-rigid registration of multi-modal images using both mutual information and cross-correlation. Med Image Anal. (2008) 12:3–15. doi: 10.1016/j.media.2007.06.005 17669679

[14] Viola P Wells WM . Alignment by maximization of mutual information. Int J Comput Vis. (1997) 24(2):137–54. doi: 10.1023/A:1007958904918

[15] Yang D Goddu SM Lu W Pechenaya OL Wu Y Deasy JO . Technical note: deformable image registration on partially matched images for radiotherapy applications. Med Phys. (2010) 37:141–5. doi: 10.1118/1.3267547 20175475 PMC4108644

[16] Haskins G Kruger U Yan P . Deep learning in medical image registration: a survey. Mach Vis Appl. (2020) 31:8. doi: 10.1007/s00138-019-01042-8

[17] Fu Y Lei Y Wang T Curran WJ Liu T Yang X . Deep learning in medical image registration: a review. Phys Med Biol. (2020) 65:20TR01. doi: 10.1088/1361-6560/ab843e 32217829 PMC7759388

[18] Lafarge MW Moeskops P Veta M Pluim JPW Eppenhof KAJ . Deformable image registration using convolutional neural networks. Proc SPIE Med Imaging. (2018) 10574:105740S. doi: 10.1117/12.2292443

[19] Jaderberg M Simonyan K Zisserman A Kavukcuoglu K . Spatial transformer networks. Adv Neural Inf Process Syst. (2015) 28:2017–25. doi: 10.48550/arXiv.1506.02025

[20] Balakrishnan G Zhao A Sabuncu MR Guttag J Dalca AV . VoxelMorph: a learning framework for deformable medical image registration. IEEE Trans Med Imaging. (2019) 38(8):1788–800. doi: 10.1109/tmi.2019.2897538 30716034

[21] Liu R Lehman J Molino P Such FP Frank E Sergeev A . An intriguing failing of convolutional neural networks and the CoordConv solution. arXiv preprint. (2018) 1807.03247. doi: 10.48550/arXiv.1807.03247

[22] Dosovitskiy A Beyer L Kolesnikov A Weissenborn D Houlsby N . An image is worth 16x16 words: transformers for image recognition at scale. arXiv preprint. (2020) 2010.11929. doi: 10.48550/arXiv.2010.11929

[23] Chen J He Y Frey EC Li Y Du Y . ViT-V-Net: vision transformer for unsupervised volumetric medical image registration. arXiv preprint. (2021) 2104.06468. doi: 10.48550/arXiv.2104.06468

[24] Zhang R Mo H Wang J Jie B He Y Jin N . UTSRMorph: a unified transformer and superresolution network for unsupervised medical image registration. Med Imaging IEEE Trans. (2025) 44:891–902. doi: 10.1109/tmi.2024.3467919 39321000

[25] Kuckertz S Bender B Modersitzki J Maier-Hein K . “ Locally normalized gradient fields for multi-modal groupwise image registration,” in: Bildverarbeitung für die Medizin 2022. (2022). Wiesbaden: Springer Vieweg, 87–92. doi: 10.1007/978-3-658-37909-5_18

[26] Maes F Collignon A Vandermeulen D Marchal G Suetens P . Multimodality image registration by maximization of mutual information. IEEE Trans Med Imaging. (1997) 16:187–98. doi: 10.1109/42.563664 9101328

[27] Chang CW Tian Z Qiu RLJ Mcginnis HS Bohannon D Patel P . Exploration of an adaptive proton therapy strategy using CBCT with the concept of digital twins. Phys Med Biol. (2025) 70:25010. doi: 10.1088/1361-6560/ada684 39761649 PMC11740008

